# The contribution of animal models to understanding the role of the immune system in human idiopathic pulmonary fibrosis

**DOI:** 10.1002/cti2.1153

**Published:** 2020-07-27

**Authors:** Tylah Miles, Gerard F Hoyne, Darryl A Knight, Mark W Fear, Steven E Mutsaers, Cecilia M Prêle

**Affiliations:** ^1^ Institute for Respiratory Health Nedlands WA Australia; ^2^ Centre for Respiratory Health School of Biomedical Sciences University of Western Australia Nedlands WA Australia; ^3^ Centre for Cell Therapy and Regenerative Medicine School of Biomedical Sciences University of Western Australia Nedlands WA Australia; ^4^ School of Health Sciences University of Notre Dame Australia Fremantle WA Australia; ^5^ Providence Health Care Research Institute Vancouver BC Canada; ^6^ University of British Columbia Vancouver BC Canada; ^7^ Burn Injury Research Unit School of Biomedical Sciences The University of Western Australia Crawley WA Australia; ^8^ Ear Science Institute Australia Nedlands WA Australia

**Keywords:** animal models, bleomycin, fibrogenesis, inflammation, innate and adaptive immune system

## Abstract

Pulmonary fibrosis occurs in a heterogeneous group of lung disorders and is characterised by an excessive deposition of extracellular matrix proteins within the pulmonary interstitium, leading to impaired gas transfer and a loss of lung function. In the past 10 years, there has been a dramatic increase in our understanding of the immune system and how it contributes to fibrogenic processes within the lung. This review will compare some of the models used to investigate the pathogenesis and treatment of pulmonary fibrosis, in particular those used to study immune cell pathogenicity in idiopathic pulmonary fibrosis, highlighting their advantages and disadvantages in dissecting human disease.

## Graphical abstract

This review will compare some of the models used to investigate the pathogenesis and treatment of pulmonary fibrosis, in particular those used to study immune cell pathogenicity in idiopathic pulmonary fibrosis, highlighting their advantages, disadvantages and suitability in representing the human disease.

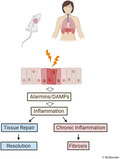

## Introduction

Idiopathic pulmonary fibrosis (IPF) is the most common of the interstitial lung diseases. It is characterised by excessive extracellular matrix (ECM) deposition in the lung with a progressive decline in lung function and a prognosis of 3–5 years after diagnosis. The disease affects men slightly more frequently than women, and the age of disease onset is from 40 to 70 years.^1^, ^2^ Seven distinct subtypes of interstitial lung disease have been proposed by the American Thoracic Society/European Respiratory Society.^3^, ^4^ Of these histopathological subtypes, usual interstitial pneumonia (UIP) is the histological pattern that characterises patients with clinical IPF.

Usual interstitial pneumonia typically demonstrates a heterogeneous appearance with areas of fibrosis within the peripheral region of the lung characterised by fibroblast proliferation, myofibroblast accumulation and excessive collagen deposition, surrounded by normal lung architecture.^5^, ^6^, ^7^ The formation of fibroblast foci is associated with a breakdown of alveolar septal walls and enlargement of airspaces, giving the appearance of ‘honeycombing’. The damage to the alveolar structure results in decreased lung compliance and inefficient gas exchange, leading eventually to respiratory failure. UIP lungs demonstrate mild inflammation with lymphocytic infiltration in the alveolar interstitium. The aetiology of the disease is unknown but genetic studies have identified a number of susceptibility genes that play important roles in epithelial cell function and the maintenance and integrity of the epithelial barrier.^8^, ^9^, ^10^, ^11^, ^12^, ^13^, ^14^ There is also growing evidence for the role of the innate and adaptive immune response in the initiation and/or progression of fibrotic diseases including IPF.^15^, ^16^, ^17^, ^18^

The underlying pathobiology of IPF was considered a chronic inflammatory immune response because of the damage of the airway epithelium.^2^, ^19^ More recently, attention has focused on intrinsic defects within the lung epithelial cells that lead to an abnormal repair process in response to damage.^6^, ^20^, ^21^ The abnormal re‐epithelialisation and/or response to epithelial damage triggers the release of various cytokines and inflammatory mediators that results in epithelial cell hypertrophy, uncontrolled fibroblast proliferation and myofibroblast accumulation, impaired clearance of (myo)fibroblasts leading to excessive ECM deposition, tissue remodelling and angiogenesis.^22^, ^23^ The inflammatory response in IPF is diverse, involving innate and adaptive lymphocytes including antibody‐producing plasma B cells that are recruited to the lung tissue environment.^15^, ^22^, ^24^, ^25^, ^26^ Lymphocytes accumulate within discrete foci that are distributed adjacent to areas of active fibrosis^26^, ^27^, ^28^ but whether these cells are crucial in disease pathogenesis of IPF is unclear. IPF does not appear to behave as a single clinical disease entity but rather as a spectrum, as evidenced by differences in expression of inflammatory markers and autoantibodies.^25^, ^29^, ^30^, ^31^, ^32^, ^33^ Acute exacerbations of IPF have been associated with immune cell infiltration of the lung but why this occurs and how it drives the profibrotic response are unknown.^34^ In this review, we examine a range of different animal models that have been used to help investigate the key immunological changes that occur in the development of lung fibrosis, and their relevance to IPF.

## Animal models of pulmonary fibrosis

Some domestic animals such as cats, dogs and horses can develop spontaneous pulmonary fibrosis, which share many of the histopathological features observed with human IPF. In dogs, the West Highland white terrier is one breed that is particularly susceptible to canine IPF.^35^ The disease shares many clinical features of human IPF with coarse crackling heard on thoracic auscultation, pulmonary hypertension and/or airway collapse. In dogs, ground glass opacification and traction bronchiectasis have been demonstrated with subpleural and peribronchiolar fibrosis, honeycombing and alveolar epithelial changes consistent with human UIP.^35^ In cats, spontaneous IPF‐like disease is associated with interstitial fibrosis with fibroblast/myofibroblast accumulation, honeycombing and type II pneumocyte hyperplasia.^36^, ^37^ Horses and donkeys can develop an IPF‐like disease but unlike cats and dogs, where the disease appears to be spontaneous, the development of equine IPF is linked to equine herpesvirus 5 infection.^38^

Genetic deficiency in mice can lead to the development of spontaneous pulmonary and age‐related lung disease.^39^, ^40^, ^41^, ^42^ More commonly, mouse models of pulmonary fibrosis have exposed mice to various agents including cytotoxic agents (e.g. bleomycin [BLM]), the delivery of profibrotic cytokines (e.g. transforming growth factor beta [TGF‐β] and interleukin [IL]‐13) or pharmacological agents (e.g. phorbol myristate acetate [PMA]). However, these induced mouse models do not follow the progression of human IPF as they often utilise young adult mice and elicit acute lung damage with inflammatory responses that lead to tissue fibrosis. The major concern is that each model relies on a specific known insult that will trigger an immune response that differs slightly in its cellular constitution and duration. Furthermore, these animal models are more representative of an acute lung injury, with fibrosis that in most cases eventually resolves, unlike the chronic, progressive and ultimately fatal disease observed with human IPF. Despite this, these models have provided valuable insight in the mechanisms leading to collagen regulation, fibrosis pathogenesis and possible treatments.

## Modelling human lung fibrosis in a mouse model

Mouse models are widely used throughout biomedical research to study disease pathogenesis of acute and chronic diseases. Ideally, an animal model should reproduce the cardinal clinical features of the human disease/condition and its symptoms. Although animal models may not replicate all aspects of the human disease, they can nevertheless provide valuable insight into the pathobiology associated with tissue damage, cellular inflammation, immune regulation, tissue repair and fibrosis, because of the high degree of conservation in immune systems and tissue structure and function.

**Figure 1:**
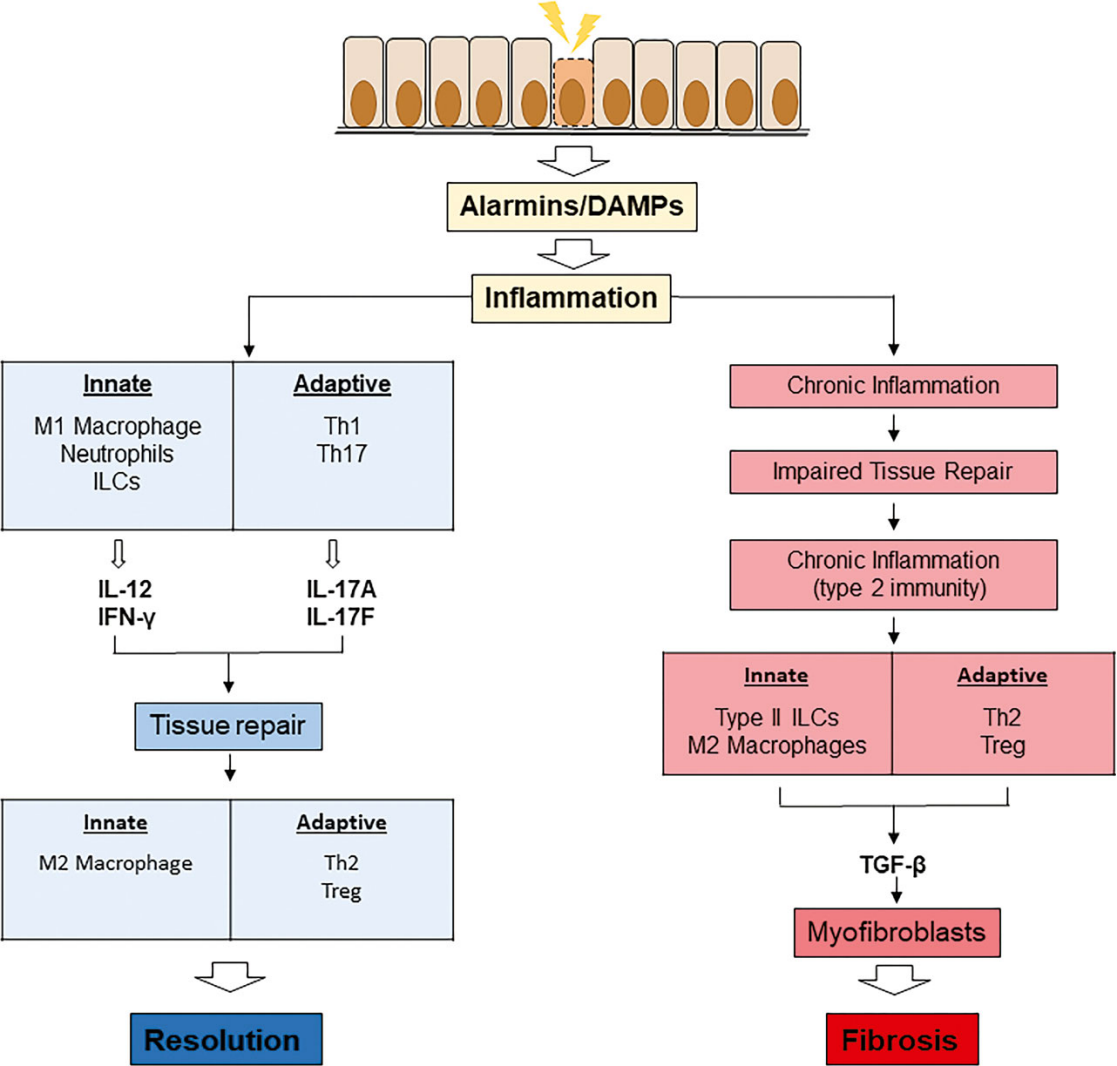
Immunological outcomes of immune response to tissue damage in the lung, leading to distinct outcomes of tissue repair or pulmonary fibrosis. Innate and adaptive immune responses are central to both tissue repair and chronic inflammatory responses that lead to tissue fibrosis. Alarmins or DAMPs are released in response to epithelial damage and cell death. These host‐derived molecules are sensed by innate leukocytes (e.g. neutrophils and macrophages or innate lymphoid cells) and help in the coordinated response of adaptive immune cells including T cells and B cells. Tissue repair follows a pattern of type 1 immunity, and this switches to a type 2 dominant immune response to promote tissue repair. In contrast, under conditions of chronic tissue damage, a dominant type 2 immune response drives both innate and adaptive immune cells that can lead to the differentiation of fibroblasts to myofibroblasts, tissue remodelling and fibrosis; a characteristic feature of IPF that is responsible for the reduction in lung function.

**Figure 2:**
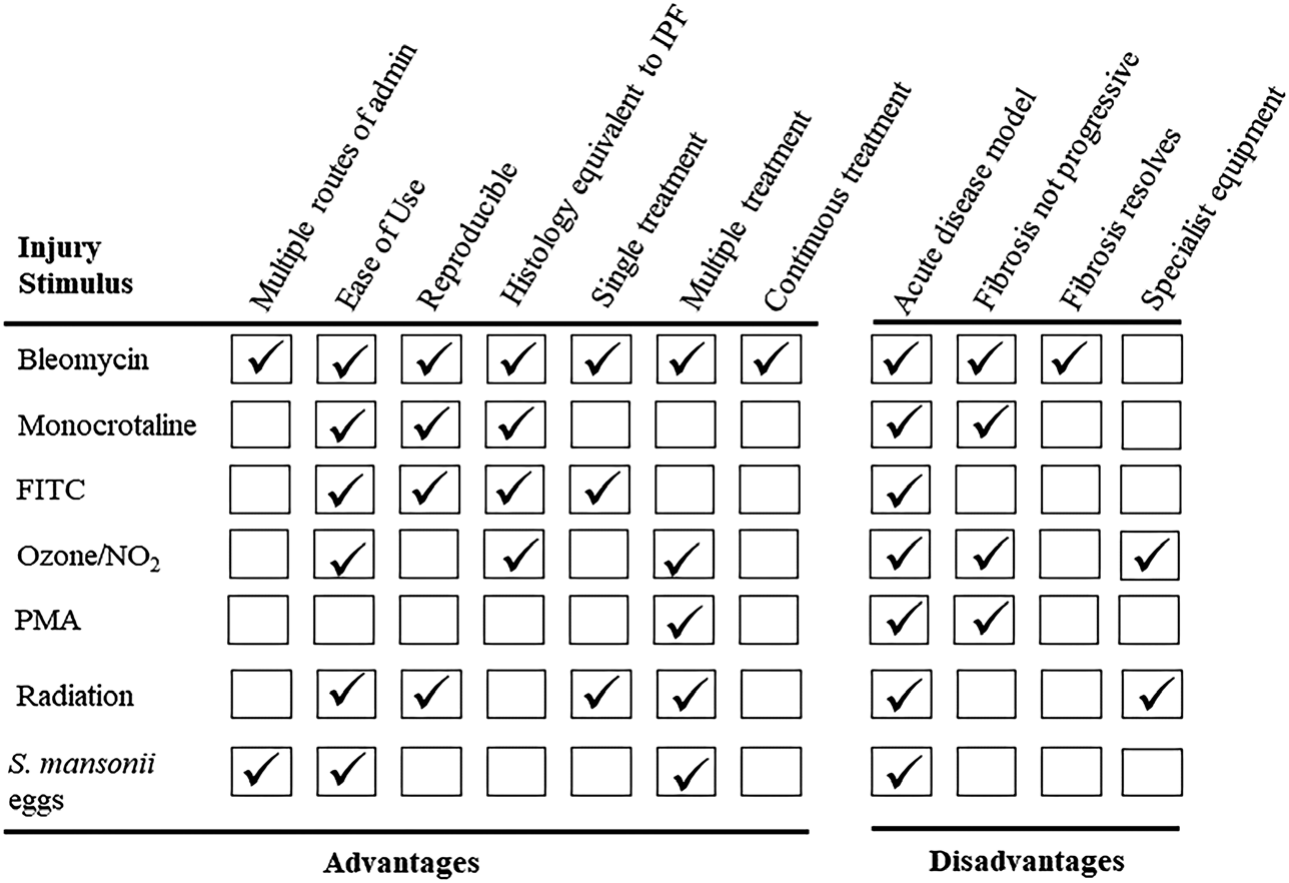
Experimental animal models of pulmonary fibrosis. A number of different treatments have been used to elicit pulmonary fibrosis in animals. There are advantages and disadvantages of the different treatments, and although none of them elicit a pathology identical to human IPF, each model recapitulates some of the key features of IPF and provides a good model to study collagen regulation in a disease setting.

Idiopathic pulmonary fibrosis in humans is thought to arise from an environmental stimulus that causes damage to the lung epithelium. Chronic exposure to a damaging stimulus may disrupt the normal tissue repair process that results in chronic inflammation and eventually leads to collagen synthesis and tissue remodelling as summarised in Figure 1. A number of different treatments have been used to elicit pulmonary fibrosis in mice (Table 1). Some have utilised known aetiological agents including asbestos, silica and radiation, while others have used chemical agents such as BLM, monocrotaline, fluorescein isothiocyanate (FITC), oxidants and PMA. The use of these generic agents and their appropriateness as models to investigate the immune response in lung fibrosis are summarised in Table 1 and Figure 2. Studies using different animal models to investigate the immunological basis of lung fibrosis will be discussed, with the focus mainly on the BLM‐induced lung fibrosis model as it is the most widely used.

**Table 1:** Animal models of pulmonary fibrosis

Inducing agent	Lung condition	References
Adenoviral‐TGF‐β	IPF	106, 107, 108, 109, 110
Asbestos	Asbestosis	111, 112, 113
Bleomycin	ARDS, IPF	69, 73, 87, 114, 115, 116, 117
Canine IPF (spontaneous)	IPF	35, 118
Feline IPF (spontaneous)	IPF	37, 118, 119
Equine IPF (spontaneous)	IPF	120
Haptens	Autoimmune‐mediated IPF	50, 51, 121
Irradiation	Radiation‐related pneumonitis	90, 122
Monocrotaline	ARDS, IPF	43, 44, 45, 46
Nanoparticles	ILD, COPD	123, 124, 125
Oxidants	ARDS, IPF	55, 56, 57, 59
Phorbol myristate acetate (PMA)	ARDS, IPF	64
Reovirus	BOOP	126
Silica	Silicosis	127, 128, 129
Welding fumes/metals	Pneumoconiosis	130

ARDS, acute respiratory distress syndrome; BOOP, bronchiolitis obliterans organising pneumonia; COPD, chronic obstructive pulmonary disease; ILD, interstitial lung disease; IPF, idiopathic pulmonary fibrosis.

### Monocrotaline

Monocrotaline (MCT) is a pyrrolizidine alkaloid that is metabolically activated by the liver and is both pneumotoxic and hepatotoxic.^43^ Subcutaneous administration of a single dose of MCT induces pulmonary hypertension and respiratory distress syndrome that resembles the human conditions both morphologically and functionally.^44^ Animals are usually euthanised 3–6 weeks after injection when the pathologies are fully manifested. The lungs show a severe inflammatory reaction with haemorrhage and oedema. Parenchymal changes include a reduced number of alveoli, thickened alveolar septa and accumulation of inflammatory cells, predominantly macrophages. Small arteries and arterioles show a reduced lumen, thickening of the wall with hyperproliferation of the media, inflammatory cells and increased collagen in the adventitia.^43^, ^44^, ^45^, ^46^ Mice treated with multiple subcutaneous injections of MCT for up to 18 weeks and then euthanised after 28 weeks from the start of treatment^46^ showed severe interstitial pneumonia and pulmonary fibrosis, with a remarkable increase in collagen deposition in alveolar septa after 8 weeks of treatment. However, the doses used in this study were highly toxic with many mice dying within the study period. In a rat model of MCT‐induced pulmonary arterial hypertension (PAH), MCT treatment induced perivascular infiltration of macrophages, mast cells and T cells which was reduced by inhibiting CXCL12, which is a key homeostatic chemokine that controls leukocyte migration into a range of tissues including the lung.^47^ Cuttica and colleagues investigated the role of T‐cell‐mediated immune pathology in MCT‐induced PAH in mice. They treated *Rag1^‐/‐^* mice, which are devoid of mature B and T cells, and found that they were protected from vascular injury. They also provided evidence for a role of CD4^+^ T cells in promoting pulmonary vascular remodelling in the same model.^48^

### Fluorescein isothiocyanate

Fluorescein isothiocyanate (FITC) is a skin‐sensitising hapten capable of inducing specific immune responses^49^ and produces fibrosis in the lungs of mice and rats following a single intratracheal instillation.^50^ An advantage of this system is that the distribution of FITC in the lung can be directly visualised. However, this agent is hard to administer as the molecule is relatively insoluble and requires sonication to give a better dispersion in the lungs with a more uniform and reproducible injury.^51^

After instillation of FITC, the animals develop a pattern of injury consistent with acute lung injury. This includes haemorrhage, alveolar wall oedema, eosinophilic alveolar exudate, a marked infiltrate of mononuclear cells and neutrophils principally around the bronchioles, and bronchial epithelial cell hyperplasia.^50^, ^51^ There is patchy focal destruction of the normal lung architecture with focal interstitial fibrosis by 21 days, which persists for at least five months with a predominantly mononuclear cell infiltrate.^50^ Both the infiltrate and the scarring were confined to peribronchial areas of FITC deposition. Christensen *et al*.^51^ examined the role of lymphocytes in FITC‐induced lung fibrosis in the different mouse strains: C57BL/6 and BALB/c. There appeared to be no difference in fibrosis onset between the two strains, suggesting that there were no background specific genes that altered pathology. It was also initially speculated that FITC‐induced pulmonary fibrosis would be driven by specific immunity to the fluorescein hapten.^50^ Six months following FITC treatment, CD3^+^ T cells and B220^+^ B cells were co‐localised within lymphoid clusters in the lungs, adjacent to areas of active tissue fibrosis,^52^ similar to the pattern observed in human IPF lung.^27^, ^28^ However, FITC‐treated T‐cell‐depleted SCID and *Rag1* ^‐/‐^ mice still developed lung fibrosis suggesting that the adaptive immune system is not involved in fibrogenesis in these animals.^51^ Interestingly, FITC treatment in the absence of the chemokine receptor CCR2, which is responsible for monocyte infiltration, reduced fibrosis, suggesting that while the adaptive immune response may not be essential, the activation of the innate immune responses may play a critical role in driving FITC‐induced lung fibrosis.^53^, ^54^

### Ozone (O_3_)/nitrogen dioxide (NO_2_)

Pulmonary fibrosis is a common result of long‐term exposure to O_3_ and NO_2_, with more pronounced fibrosis when the gases are mixed.^55^ Rats exposed to a mixture of O_3_ and NO_2_ for 6 h/day demonstrated a triphasic pattern of lesion development. Lesions developed over the first 3 weeks of exposure then partially resolved during the middle three weeks before progressing in severity in the final three weeks, with a significant increase in collagen deposition.^55^, ^56^ This finding was confirmed when half the concentration of O_3_ and NO_2_ was used but with continuous exposure.^57^ Histological changes were mild compared with the higher oxidant concentration, despite a twofold increase in the cumulative dose. This showed that pulmonary fibrosis is more dependent on the concentration of oxidants than the cumulative dose. Both models showed some of the notable pathologic features of end‐stage IPF in humans, including severely distorted alveolar structure, increased deposition of collagen, collections of mast cells, obliteration of air spaces in fibrotic regions and focal areas of enlarged abnormal air spaces. However, the distribution of fibrosis was primarily in the centriacinar regions of the lung whereas UIP demonstrates a more peripheral fibrosis.^2^, ^58^ Furthermore, this response was only seen in the presence of a continuing insult, whereas in IPF, progression occurs in the apparent absence of the inciting agent.

A single exposure to O_3_ can induce desquamation of lung epithelial cells with concomitant secretion of chemokines CXCL1, CCL2/MIP1 and IL‐6, which promote macrophage and neutrophil recruitment to the site of damaged lung tissue.^59^ Antibody depletion of neutrophils reduced inflammation and parenchymal injury, which was associated with increased levels of amphiregulin, a cytokine secreted by type 2 ILCs and CD4^+^ Tregs to promote tissue repair.^60^, ^61^ Ozone exposure elicited increased expression of type 2 cytokines IL‐4, IL‐5, IL‐13, eotaxin and monocyte chemoattractant protein (MCP)‐2 (also known as CCL8) mRNA in wild‐type C57BL/6 and *Rag2* ^‐/‐^ mice.^62^ In contrast, ozone‐exposed ILC‐deficient *Rag2* ^‐/‐^/*Il2rg* ^‐/‐^ mice had no nasal lesions or overexpression of Th2‐ or ILC2‐related transcripts.^62^ In a similar manner, Michaudel *et al*. used this model to examine the role of IL‐33 cytokine signalling in a model of O_3_‐induced airway hyper‐responsiveness. The authors determined that O_3_ exposure led to an immediate increase in IL‐33 which was followed by increased numbers of neutrophils and alveolar macrophages within 6–18 h post‐O_3_ treatment, while lymphocyte and eosinophil numbers were increased by 18–24 h post‐treatment. Genetic deletion of IL‐33 (*Il‐33* ^‐/‐^) or its receptor ST2 (also known as IL‐1RL1) (*St2* ^‐/‐^) in mice that were exposed to a single dose of O_3_ displayed exacerbated epithelial injury and neutrophilic inflammation. Enhanced neutrophilic inflammation mediated via O_3_ treatment was also observed following anti‐IL‐33R blockade.^63^ Conversely, the administration of IL‐33 protein to *IL33* ^‐/‐^ mice reduced neutrophil recruitment in response to O_3_ exposure. These data suggest that the IL‐33 signalling pathway may play an important role in the regulation of lung homeostasis and in particular to promote the maintenance of the epithelial barrier.^63^

### Phorbol myristate acetate

Phorbol myristate acetate (PMA) is a potent activator of leukocytes including neutrophils, macrophages, lymphocytes and platelets. A single intravenous dose of PMA in rabbits induced an acute haemorrhagic pneumonitis followed by a phase of interstitial inflammation involving neutrophils and macrophages.^64^ Progressive interstitial fibrosis did not occur, and the lungs were virtually normal after 14 days. The development of histologically determined fibrosis required the continued administration of PMA (5 of every 7 days) in this model. Although the amount of fibrosis increased with the number of PMA injections, the amount of interstitial inflammation decreased, suggesting that the chronic phase of the lung reaction is not simply the consequence of acute lung injury, which is more consistent with human IPF than the other models described.

### Radiation‐induced pulmonary fibrosis

Thoracic exposure to radiation in humans can induce strong inflammatory responses that lead to alveolitis or even fibrosing alveolitis. Radiation‐induced pulmonary fibrosis is characterised by tissue damage, epithelial and fibroblast cell proliferation and remodelling of the lung interstitium. To mimic the pathologic changes that occur with radiation exposure in humans, mice were exposed to a single 18 Gy dose of radiation. In this model, lung fibrosis developed within 6 months of treatment. Fox and colleagues exposed C57BL/6, A/J and C3HeJ strains of mice to a single full thoracic exposure of 18 Gy of radiation and observed that C57BL/6 mice were most susceptible to developing lung fibrosis compared to the other strains.^65^ However, compared to the other lung fibrosis models discussed, the radiation model is time consuming and requires a high degree of monitoring as exposure to irradiation increases the susceptibility to lymphopenia and thus predisposes the animals to infection.

### Schistosoma mansoni eggs

Chronic infection with the blood fluke *Schistosoma mansoni* causes a severe pathology including liver fibrosis and splenomegaly. The fibrotic response is caused by an immune response to the parasite eggs rather than the parasite itself. The parasite egg antigens induce a delayed type hypersensitivity response characterised by a CD4^+^ Th2 immune response with production of type 2 cytokines IL‐4, IL‐5 and IL‐13, a rise in IgE antibody levels, activation of alternative macrophages and the recruitment of eosinophils.^66^ Following intraperitoneal sensitisation and intravenous challenge, *S. mansoni* eggs are transported to the lung via the pulmonary arteries where they become trapped within the lung parenchyma with subsequent formation of granulomas composed of lymphocytes, eosinophils and alternatively activated macrophages. These granulomas are associated with inflammation in the broncho‐alveolar spaces, expansion of the draining lymph nodes and CD4^+^ T‐cell activation which in turn can lead to pulmonary fibrosis.^66^ More recently, activation and recruitment of ILC2 cells have been shown to contribute to the pulmonary fibrosis in response to *S. mansoni* egg sensitisation through secretion of IL‐25.^66^ Hams and colleagues showed that antibody‐mediated depletion of ILC2 cells in lymphocyte‐deficient *Rag1* ^‐/‐^ mice could reduce the size of *S. mansoni* egg‐induced granulomas and the degree of pulmonary fibrosis.^66^ This result indicates that pulmonary fibrosis in this disease model does not rely on CD4^+^ T cells but emphasises an important role for the innate immune response to orchestrate the lung fibrotic response. To relate these findings in mice back to human IPF, the authors identified increased pulmonary expression of IL‐25 and recruitment of ILC2 cells to the lung of IPF patients.^66^

### Bleomycin (BLM)

The best characterised and most widely used agent to induce pulmonary fibrosis in mice and rats is BLM, an anti‐cancer drug that induces DNA damage within target cells.^67^, ^68^ BLM is usually administered in saline or PBS as a single dose via intratracheal, intranasal, intraperitoneal, oropharyngeal or intravenous routes; the concentration administered depending on the route of administration and the species and strain of animal used.

Following a single BLM challenge, the animals often experience weight loss within the first few days, which is associated with the acute lung injury. After 5–7 days, weights begin to increase and animals eat and behave normally. The lung weight is usually maximal at about 7 days after BLM treatment.^69^ The histologic pattern of lung injury is similar for all species but does vary slightly depending on the route of administration.^70^, ^71^, ^72^ The initial injury is predominantly focused around bronchioles, with areas of microvascular leakage and early hyperplastic changes in type II pneumocytes in regions of inflammatory cell influx. By day 7 following BLM treatment, the injury is more widespread and involves the distal lung parenchyma with multiple inflammatory foci and oedema present within alveolar septa. By day 14, the lungs show a more mature regional interstitial fibrosis with an increase in macrophages and focal lymphocytosis and lymphoid expansion. Focal alveolar re‐epithelialisation is present with extensive collagen deposition and remodelling of the alveolar unit, and there is often a low‐grade focal ‘honeycombing’ reaction present, consistent with emphysematous changes. By days 21–30, there is evidence of focal condensation of ECM. Macrophage and lymphocyte margination is marked, particularly within the periphery of the fibrotic areas, and regional re‐epithelialisation of the alveolar septa is pronounced. By 120 days, there is bronchiolar and peribronchiolar fibrosis, together with inflammation and emphysematous changes, with large areas of normal lung. These changes are consistent across studies and are thought to reflect similar changes observed with IPF.^70^, ^72^ Intravenous or intraperitoneal administration of BLM gives a similar pattern of lung fibrosis although the initial site of injury is the endothelium of capillaries and larger vessels and the perivascular lung structures of the subpleural parenchyma.^73^ Aran and colleagues have used single‐cell RNA sequencing (scRNAseq) to characterise the heterogeneity of macrophages following BLM‐induced fibrosis in mice. They identified a putative profibrogenic macrophage population that displayed a signature gene profile (*CX3CR1* ^+^, *CCR2* ^+^, *MHCII* ^+^), intermediate between a monocyte‐derived macrophage and alveolar macrophage, that produced high levels of platelet‐derived growth factor AA that directs fibroblast proliferation.^74^ Depletion of CXCR3^+^ macrophages in mice from day 8 following BLM administration revealed a significant reduction in SiglecF^+^ macrophages and fibroblasts in the lung and decreased collagen synthesis. These findings suggest an important role for CX3CR1^+^ macrophages in lung fibrosis. Furthermore, they were able to demonstrate the existence of a similar population of transitional macrophages within lung samples from human IPF patients.^74^ Reyfman and colleagues used RNAseq to analyse lung biopsy samples from IPF patients, compared to healthy lung tissue obtained from transplant donors. They compared scRNAseq data sets obtained from immune cells, epithelial cells and fibroblasts. They identified a similar novel profibrogenic macrophage population in IPF patients and were able to establish a single‐cell atlas of pulmonary fibrosis.^22^ This novel and unique resource revealed for the first time a significant level of heterogeneity of alveolar macrophages and lung epithelial cells within IPF patients.

A continuous or repetitive delivery method of BLM appears to produce more fibrosis in the lung, and a fibrotic phenotype more closely resembling IPF than the single BLM delivery method.^73^, ^74^, ^75^, ^76^, ^77^ Heterogeneous areas of inflammation and fibrosis, with persistent deposition of collagen and collapse of alveolar structures, likely leading to reduced lung function 5–6 weeks after BLM exposure, support the progressive nature of the pulmonary lesion in this model, thereby more closely resembling the human UIP pattern.^69^, ^73^, ^78^ Aged mice have also been used in different models of fibrosis, including BLM‐induced lung fibrosis, to try and more closely resemble human IPF.^79^, ^80^, ^81^ However, there is limited information on the effect of aging on the immune response within these models.

‘Humanised’ immunodeficient mice have proven a valuable addition to study the role of human cells/tissues in disease pathobiology associated with lung fibrosis. These studies have been restricted to the transfer of human IPF versus control lung fibroblasts to investigate the profibrotic potential of these mesenchymal cells. Two recipient mouse strains have been used that included the C.B.‐17 SCID/beige^82^, ^83^ and the NOD‐scid‐IL2Rγc^–/–^ [NSG] mouse strain.^84^ Pierce *et al*. first demonstrated that human IPF fibroblasts but not control fibroblasts could drive pulmonary fibrosis in recipient mice that elicited a disease pathology similar to that observed in human IPF patients. Furthermore, they targeted human CCR7 or CCL21 proteins using specific antibodies to neutralise the respective protein and showed that the immunotherapy could attenuate the progression of lung fibrosis in mice.^83^ Jones *et al*.^82^ investigated the role of the MAP3K19 enzyme that has been shown to be upregulated in IPF patients. They transferred cultured human IPF fibroblasts into C.B‐17SCID/bg mice and showed that targeting the MAP3K19 enzyme with either siRNA or a small molecule inhibitor could attenuate lung fibrosis in recipient mice.^82^ Geng *et al*.^84^ also used the humanised mouse model to identify two distinct fibroblast populations from IPF lung based on the expression of the check point molecule programmed death ligand‐1 (PDL‐1, also called CD274). PD‐L1^+^ fibroblasts demonstrated greater motility and invasive properties compared to PD‐L1^‐^ fibroblasts. NSG mice that received PDL1^+^ cells developed pulmonary fibrosis to a greater extent than NSG mice that received PDL1^‐^ fibroblasts. Furthermore, they showed that antibody‐mediated targeting of PD‐L1 or CRISPR‐mediated depletion of PD‐L1 both reduced the level of pulmonary fibrosis in NSG recipient mice.^84^ Habiel and colleagues have suggested a role for CD28null T cells in the pathogenesis of lung fibrosis by transferring T cells from an IPF patient to a NSG mouse. The mice experienced unresolved lung remodelling 63–65 days after injection. The authors suggest that this is due to injury to type II alveolar epithelial cells, shown by loss in BAL surfactant protein C.^85^

It is also necessary to consider the selection of the appropriate mouse strain, as there is strong evidence that the genetic background can influence the degree of lung fibrosis following BLM treatment.^86^ C57BL/6J mice are the most commonly used strain for BLM treatment, because of the reproducibly high levels of inducible lung collagen deposition, that are maintained for at least 12 weeks. It has been reported that other inbred strains such as DBA/2 show more prolonged fibrosis in multiple exposure studies.^87^ In contrast, inbred stains such as A/J, C3Hf/KAM or C3H/HeJ are protected from BLM‐induced fibrosis.^88^, ^89^, ^90^ This disparity in the fibrotic response between mouse strains prompted scientists to examine whether there may be BLM susceptibility genes for lung fibrosis. Using a series of genetic crosses, a region on chromosome 17 named the *bleomycin‐induced pulmonary fibrosis 1* (BLMpf1) locus was identified.^90^, ^91^ The locus was further refined down to a 0.71 Mb region by using subcongenic mice that contained 17 C3H/HeJ alleles in the BLMpf1interval on a C57BL/6J background.^92^

## Identifying genes that promote susceptibility to BLM‐induced pulmonary fibrosis

The BLMpf1 interval contains 40 known proteins and 17 of the genes contain single nucleotide polymorphisms (SNPs) that affect the encoded protein. Some of the key proteins within the interval, which showed linkage to fibrotic disease in 23 inbred mouse strains, were Butyrophilin‐like (BTNL)6, activating transcription factor (ATF)6, Notch4, Tenascin XB and complement components 4a (C4a) and C4b.^90^ Although the protective alleles led to an increase in macrophage numbers in BAL fluid in response to BLM treatment compared to the wild‐type mice, subcongenic mice showed a significantly reduced number of lymphocytes in broncho‐alveolar lavage (BAL) compared to wild‐type C57BL/6J mice. Likewise, examination of gene expression in lung epithelial cells in response to BLM treatment *in vivo* showed a significant increase in expression of two butyrophilin‐like molecules BTLN4, BTLN6 and the complement protein C4b.^92^

## Butyrophilin‐like proteins

The butyrophilins (BTN) and BTNL genes are part of the immunoglobulin superfamily. The BTNL family comprises six mouse and five human genes.^93^ They are structurally related to the costimulatory proteins CD80 (CD80.CD86, ICOS) and the inhibitory molecule PD‐L1, and they are evolutionarily related to major histocompatibility complex (MHC) molecules. The BTN family is composed of two mouse and six human genes.^93^ Although the function of BTN/BTNL genes remains poorly characterised, recent studies have indicated a role in immune regulation of TCRγδ+ cell subsets within both mouse TCR Vγ7+ and human TCRVγ4 cells within intestinal epithelium.^93^ Human BTN3A1 directs blood TCRγδ cells to low molecular weight microbial and endogenous metabolites, but it is not known if this requires recognition of TCR‐BTN3A1 binding. In addition, Di Marco Barros and colleagues recently demonstrated that intestinal epithelial cells can express BTNL molecules and these are used to activate TCRγδ cells via their TCR, and this can shape the local TCRγδ compartment in the gut.^93^ The BTNL1, 2, 4 and 6 genes were identified within the *BLMpf1* interval. Of those, BTNL1 and 4 were shown to be expressed on lung epithelium of C57BL/6J mice but it was only the BTNL6 gene which was identified in a genomewide association study (GWAS) to significantly associate with fibrotic lung disease in C57BL/6J mice.^90^ Given the emerging role of BTNL proteins and TCRγδ cells in human and mouse mucosal tissue homeostasis,^93^, ^94^, ^95^, ^96^ it will be interesting to determine how these molecules might function in the human lung as well as in diseases such as IPF. The restricted expression of BTNL molecules may be used to sustain cognate intra‐epithelial lymphocytes in the gut, and by extension, they may help to orient lymphocytes to their correct anatomical location and to the health status of the tissue. Therefore, BTNL molecules may have a broader function outside of the gut to enable resting epithelial cells and innate T cells to interact, and this could facilitate tissue surveillance by TCRγδ cells at mucosal surfaces which play a critical role in immune stress surveillance.^93^

There is an emergence of subsets of other innate‐like T‐cell populations in the peripheral immune system, including invariant NK T cells (iNKT), mucosal‐associated invariant T (MAIT) cells and germline‐encoded mycolyl lipid‐reactive (GEM) T cells that express TCRαβ receptors with a characteristically restricted TCRV gene usage.^97^, ^98^ These innate T‐cell populations do not recognise peptide antigens but instead recognise a range of microbial products from lipids, vitamins or glycolipids in the context of non‐classical MHC proteins.^99^ These cells are believed to play an important role together with TCRγδ cells in mucosal immune surveillance. TCRγδ^+^ cells have been linked to an immunoregulatory role at mucosal surfaces for some time. McMenamin and colleagues first described an immunoregulatory role for CD8^+^ TCRγδ^+^ cells in the murine lung in response to the aerosolised protein antigen ovalbumin that were important to control IgE responses and allergic sensitisation.^100^ As the understanding of the innate and adaptive immune response has grown, we are on the verge of unravelling one of the key roles that innate immune cells play in the control of pulmonary fibrosis. This could be explored not from just a cellular level, but with the power of genomics, it will be possible to elucidate the response of different cell subsets during pulmonary fibrosis at a single‐cell resolution. The increasing number of scRNAseq studies in IPF is allowing us to identify specific immune cell populations that can be manipulated in animal models to determine their clinical relevance.

Genome editing using clustered regularly interspaced short palindromic repeats (CRISPR)/Cas9 technology has provided scientists with the ability to evaluate the role of candidate genes in disease specific processes *in vivo*. CRISPR/Cas9 editing can be used to either delete a specific target gene or alternatively, to introduce specific point mutations of a clinically relevant candidate gene. The benefit of this approach is that it could give rise to novel animal models that could be more ‘clinically relevant’ as lung fibrosis may occur spontaneously without the need of intervening agents. This approach has been applied to the study of tissue fibrosis more broadly in the heart and cystic fibrosis in the lung. Galectin 3 has been associated with pulmonary hypertension and fibrosis, and Barman and colleagues have used CRISPR‐mediated deletion of Gal3 to examine its role in pulmonary hypertension in rats.^101^ McCarron *et al*.^102^ used CRISPR/Cas9‐mediated editing in rats to generate two separate strains with either a deletion of the CFTR gene or to generate a CFTR disease‐specific allele (Phe508 del) to evaluate the role of both mutations in causing cystic fibrosis *in vivo*. A number of gene variants have been identified in IPF patients that were identified through GWAS. Some variants are more specifically related to the epithelial function,^9^, ^11^, ^13^, ^18^ while a number of variants have been associated with the TERT/PARN pathway that regulates telomere length in cells.^8^, ^103^ Disease‐specific alleles of the *Muc5b* ^9^, ^104^ gene have been evaluated using CRSPR‐mediated gene editing in human airway epithelial cells *in vitro*.^105^ The next step would be to determine whether gene editing could be used to validate whether one of these alleles could drive spontaneous pulmonary fibrosis in mice, and thus provide a clinically relevant animal model of IPF where pre‐clinical therapeutic interventions could be evaluated.

## Conclusions

Developing appropriate models to reflect human chronic disease is a challenge, particularly in diseases such as IPF where the aetiology of the disease is unknown, and the disease has likely developed over a long period of time. Many different animal models are currently used to study pulmonary fibrosis, and each model has its particular strengths and weaknesses. For this reason, care must be taken when extrapolating data from any single animal model to the human disease.

Use of animal models has led to a major growth in our understanding of the innate immune system and how it coordinates adaptive immune responses. The development of single‐cell technologies to examine gene expression and epigenetic changes, combined with the ability to analyse cells with multicolour flow cytometry and cell sorting capacity, is helping to identify different cell subpopulations within different normal and diseased tissues that have not been previously possible. It is hoped that these types of studies in animal models will inspire further research to better understand the pathogenesis of IPF.

## Conflict of interest

The authors declare no conflict of interest.

## Author contributions

**Tylah Miles:** Visualization; Writing‐original draft; Writing‐review & editing. **Gerard Hoyne:** Conceptualization; Supervision; Visualization; Writing‐original draft; Writing‐review & editing. **Darryl Knight:** Writing‐review & editing. **Mark Fear:** Supervision; Writing‐review & editing. **Steven Mutsaers:** Conceptualization; Visualization; Writing‐original draft; Writing‐review & editing. **Cecilia Prele:** Conceptualization; Supervision; Visualization; Writing‐original draft; Writing‐review & editing.
